# Long-term outcomes of non-metastatic breast cancer patients by molecular subtypes

**DOI:** 10.1186/s12905-022-01846-3

**Published:** 2022-07-04

**Authors:** Afsaneh Fendereski, Ebrahim Hajizadeh, Shahpar Haghighat, Aliakbar Rasekhi

**Affiliations:** 1 Department of Biostatistics, Faculty of Medical Sciences, Tarbiat Modares University, Tehran, Iran; 2 Breast Cancer Research Center, Motamed Cancer Institute, ACECR, Tehran, Iran

**Keywords:** Breast cancer, Molecular subtypes, Disease-free survival, Mixture cure model

## Abstract

**Background:**

Today, with the progress of medical sciences, increasing the cure probability and survival time is an important goal of cancer treatment. This study compared long-term disease-free survival (DFS) of non-metastatic breast cancer patients based on different molecular subtypes.

**Methods:**

This retrospective cohort study consisted of 1287 patients diagnosed with breast cancer and treated at Motamed Cancer Institute from 2000 to 2016 and followed up until 2018. Kaplan–Meier curve was fitted to data based on molecular subtypes. Then the semi-parametric mixture cure model was applied to determine the survival and cure probability of molecular subtypes by adjusting clinical and demographic factors.

**Results:**

Among 1287 breast cancer patients, 200 (15.5%) cases died. The mean age of patients was 47.00 ± 10.72 years. Women with the HR+/HER2-subtype had the best 5-year survival rate (84.2%), whereas other subtypes had a lower rate as follows: HR+/HER2+ (77.3%), triple-negative (76.5%), and HR−/HER2+ (62.3%). Kaplan–Meier curve calculated a cure rate of about 60% and patients who survived more than 150 months were intuitively considered cured. After adjustment for clinical and demographic variables, the cure probability of HR−/Her2+ patients was substantially lower than HR+/HER2– patients (OR = 0.22), though there were no significant variations in short-term DFS based on molecular subtypes (HR = 0.91).

**Conclusions:**

Our results confirm that the most prevalent breast cancer was HR+/HER2− tumor type which had the best prognosis. It is also concluded that HR−/HER2+ patients had the worst outcomes, with the highest rates of recurrence and metastasis and the lowest overall and disease-free survival rates.

## Background

Breast cancer is the main cause of cancer mortality and morbidity among women [1]. In 2020 over two million women were diagnosed with breast cancer worldwide [2]. According to the World Health Organization report, breast cancer was the most common cancer among Iranians in 2018 [3]. The age standardized ratio of breast cancer in Iran was 35.7 per 100,000 people in 2016, and the number is expected to increase 63% by 2025 [4]. Besides, the incidence age of breast cancer in Iran reported 10 years lower than the rest of the world, so it is suggested to start screening of women at earlier ages [5, 6].

Based on RNA sequencing analysis and immunohistochemistry studies, breast cancer is routinely clustered into several molecular subtypes as luminal A, luminal B, her2-enriched, and basal types [7, 8]. Since Ki-67 and cytokeratin testing are not commonly reported in many countries, the breast cancer subtypes are typically classified based on the presence of estrogen receptors (ER), progesterone receptors (PR), and human epidermal growth factor receptor-2 (HER-2). According to the Hormone Receptors (HR: ER and/or PR) and HER2 status, there are four main molecular subtypes including HR+/HER2+, HR+/HER2–, HR−/HER2+ and HR−/HER2− [9]. Studies have shown that biological and clinical characteristics of breast cancer patients, such as risk factors, recurrence and metastasis risk, and survival outcomes, are associated with molecular subtypes [10, 11]. As an important prognostic factor for the survival of patients with breast cancer, this classification guides systemic therapy [12]. Several studies were performed to assess the prognostic effect of biomarkers on breast cancer survival; however, the results vary in different countries, and the evidence for predicting the impacts of subtypes in developing countries is limited.

Most of common survival models assume that all subjects will eventually experience the desired event (such as recurrence, metastasis, or death) [13]. While nowadays, due to cancer treatment progress, a fraction of patients may never experience the event. These patients are cured or long-term survivors, and will have similar survival to the general population [14].

Cured patients survive longer than uncured patients and have a better quality of life, because they no longer suffer from complications. Therefore, in cancer therapy, the cure of cancer is more important goal than prolonging patients' survival time. Furthermore, the presence of cured patients in survival models increases censoring, leading to overestimated survival rate. Cure models are a professional method in survival analysis that can be used in these cases [15, 16]. The cure model estimates the odds of cure or long-term survival of patients and the survival rate of uncured patients or short-term survival. Moreover, factors related to patients' survival or cure probability can be calculated by this model. So, it can give us a better insight about factors affecting the patients' survival. In his study we aimed to compare the demographic and clinical characteristics, survival rates, and cure probability of stages I-III breast cancer patients between different molecular subtypes.

## Methods

### Sample selection

The current retrospective cohort study was performed on 1287 female diagnosed with breast cancer admitted at Motamed Cancer Institute, Tehran, Iran. Women with pathologically confirmed primary invasive breast cancer which underwent surgery from 2000 to 2016 were enrolled in the study and followed until 2018. To register the survival status of patients, they were followed up as complete as possible with mail out/phone contact, or they were referred for in person visit in clinic. If the patient had died by the time of contact, information was provided by available family members. This analysis was limited to women aged > 20, and diagnosed with stages I-IIIC (AJCC TNM staging) breast cancer. Patients with primary metastatic breast cancer, missing more than one variable and missing information on hormonal receptors or HER-2 status were excluded. Individuals who were alive at the end of the study or whose final status information was not available and could not be contacted (who withdrew) were considered censored. The study was approved by the Tarbiat Modares University Ethics Committee, Tehran, Iran (code number: IR.MODARES.REC.1397.278).

### Prognostic factors

Several variables were selected and analyzed based on the expert physicians' opinions and literature review. The variables included age at diagnosis, body mass index (BMI), education level**,** marital status, family history of cancer (any type of cancer in first- or second-degree relatives), menopausal status, pathology type, tumor size, histologic grade, stage at diagnosis (Breast Cancer Adjusted AJCC Cancer Staging Manual 6th Edition), lymph node status, ER, PR, HER2, type of surgery, hormone therapy, radiotherapy, adjuvant chemotherapy, recurrence status (local and/or contralateral recurrence), and distant metastasis status.

Since Ki-67 had not been reported in many immunohistochemical reports, we categorized breast cancer into four molecular subtypes based on HR and HER2 status, including HR-positive/Her2-positive; HR-positive/Her2-negative; HR-negative/Her2-positive, and HR-negative/Her2-negative (triple-negative). The molecular subtypes were defined by immunohistochemical staining against HR (ER and/or PR) and HER2 markers. ER and/or PR positive tumor cells ≥ 1% was defined as positive, HER2 0/1 was defined as negative, HER2 3+ defined as positive, and HER2 2+ was determined either negative or positive by fluorescence in situ hybridization (FISH).

This study contains two kinds of outcomes: survival time and cure status. For the survival time, disease-free survival (DFS) defined months from the diagnosis date to the recurrence, metastasis, death, or last follow-up; while overall survival (OS) considered as the time from diagnosis to death from any cause or last follow-up. The second outcome is cure status of patients. This is a latent variable that is specified by the model. Intuitively, the cure fraction can be estimated by Kaplan–Meier curve. We first determined the cure time of the patients using this curve. The patients who survived longer than this time were identified as cured or long-term survivors and the others are uncured or short-term survivors.

### Statistical analysis

The variables were described using frequency, mean and standard deviation. The proportion of clinicopathologic and therapeutic regimens was compared among different molecular subtypes using the chi-square test. Then survival plot was fitted by the Kaplan–Meier curve and the time of cure and cure fraction were determined. Kaplan–Meier DFS curve for stage I-III patients was prepared according to tumor subtype and compared with log-rank tests. Finally, univariate and multivariate mixture cure model performed to investigate the impact of various factors on DFS.

Mixture cure model is an extension of cox proportional hazards model that suppose the population is a mixture of two groups: the cured or long-term survivor patients and uncured or short-term survivors. Logistic regression modeled cure fraction and its related factors, and the chance of cure was indicated by the odds ratio (OR). In the survival part, factors associated with the survival time were investigated by cox model, and the risk of experiencing the outcomes was denoted by hazard ratio (HR). The Kaplan–Meier curve determined the cure fraction and adequacy of the cure model. If the tail of the curve does not reach zero and has a long and stable plateau with heavy censoring, it will be evidence for the existence of cured patients and the adequacy of the follow-up period. All calculations were performed using SAS version 9.4 and R version 4.1.1 software at a significance level of 0.05.

## Results

### Baseline characteristics

A total of 1287 women with non-metastatic breast cancer aged 21 to 87 years have entered the study, of whom 200 patients (15.5%) died, and 1087 cases (84.5%) were censored. The mean age of patients was 47.00 ± 10.72 years, 62% of patients were < 50 and 25.9% were < 40 years old. The characteristics of patients and differences in clinicopathologic and treatment features grouped by various molecular subtypes have been summarized in Table 1. In 64% of cases, the lymph nodes showed a positive result and in 68%, patients were diagnosed in early-stages (stages I and II). Patients with available chemotherapy information included 842 patients received adjuvant chemotherapy, 259 patients received Neoadjuvant chemotherapy, 99 patients received both, and 8 patients received no chemotherapy. So, we divided patients into those received and those not received adjuvant chemotherapy. All of the patients underwent surgery, and most patients received adjuvant chemotherapy (73%), radiotherapy (90%), and hormone therapy (80%). Proportions of patients by tumor subtype were 20%, 52%, 12% and 16% for HR+/Her2+, HR+/Her2−, HR−/Her2+ and HR−/Her2− respectively.

**Table 1:** Demographic, clinicopathologic and treatment features of breast cancer patients by molecular subtypes

Variables	N (%) n = 1287 (100%)	Subtype				*p*-value
		HR+/Her2 n = 261 (20.3%)	HR+/Her2− n = 658 (51.1%)	HR−/Her2 + n = 163 (12.7%)	HR−/Her2− n = 205 (15.9%)	
*Age*						
< 50	799 (62.1)	165 (63.2)	406 (61.7)	93 (57.1)	135 (65.9)	0.366
≥ 50	488 (37.9)	96 (36.8)	252 (38.3)	70 (42.9)	70 (34.1)	
*BMI*						
< 30	898 (69.8)	195 (74.7)	448 (68.1)	110 (67.5)	145 (70.7)	0.221
≥ 30	389 (30.2)	66 (25.3)	210 (31.9)	53 (32.5)	60 (29.3)	
*Education level*						
< Diploma	610 (47.4)	109 (41.8)	319 (48.5)	75 (46.0)	107 (52.2)	0.130
≥ Diploma	677 (52.6)	152 (58.2)	339 (51.5)	88 (54.0)	98 (47.8)	
*Marital status*						
Single	240 (18.6)	49 (18.8)	121 (18.4)	36 (22.1)	34 (16.6)	0.598
Married	1047 (81.4)	212 (81.2)	537 (81.6)	127 (77.9)	171 (83.4)	
*Family history of cancer*						
No	1114 (86.6)	233 (89.3)	564 (85.7)	145 (89.0)	172 (83.9)	0.250
Yes	173 (13.4)	28 (10.7)	94 (14.3)	18 (11.0)	33 (16.1)	
*Menopausal status*						
Premenopausal	836 (65.0)	172 (65.9)	432 (65.7)	92 (56.4)	140 (68.3)	0.092
Postmenopausal	451 (35.0)	89 (34.1)	226 (34.3)	71 (43.6)	65 (31.7)	
*Pathology*						
Others	149 (11.6)	24 (9.2)	78 (11.9)	23 (14.1)	24 (11.7)	0.472
IDC	1138 (88.4)	237 (90.8)	580 (88.1)	140 (85.9)	181 (88.3)	
*Tumor size (cm)*						
< 2	302 (23.5)	51 (19.5)	180 (27.4)	31 (19.0)	40 (19.5)	0.001
2–5	696 (54.1)	147 (56.3)	355 (54.0)	78 (47.9)	116 (56.6)	
> 5	289 (22.5)	63 (24.1)	123 (18.7)	54 (33.1)	49 (23.9)	
*Histological grade differentiate*						
Well	151 (11.7)	28 (10.7)	109 (16.6)	5 (3.1)	11 (5.4)	< 0.001
Moderate	779 (60.5)	159 (60.9)	423 (64.3)	88 (54.0)	107 (52.2)	
Poor	300 (23.3)	59 (22.6)	99 (15.0)	63 (38.7)	79 (38.5)	
Unknown	57 (4.4)	15 (5.7)	27 (4.1)	7 (4.3)	8 (3.9)	
*Stage*						
I	195 (15.2)	31 (11.9)	114 (17.3)	17 (10.4)	33 (16.1)	0.038
II	681 (52.9)	135 (51.7)	351 (53.3)	82 (50.3)	113 (55.1)	
III	411 (31.9)	95 (36.4)	193 (29.3)	64 (39.3)	59 (28.8)	
*Lymph node metastasis*						
No	468 (36.4)	80 (30.7)	244 (37.1)	43 (26.4)	101 (49.3)	< 0.001
Yes	819 (63.6)	181 (69.3)	414 (62.9)	120 (73.6)	104 (50.7)	
*Type of surgery*						
MRM	658 (51.1)	138 (52.9)	329 (50.0)	104 (63.8)	87 (42.4)	0.001
BCS	629 (48.9)	123 (47.1)	329 (50.0)	59 (36.2)	118 (57.6)	
Hormone therapy^*^						
No	203 (15.8)	5 (1.9)	7 (1.1)	86 (52.8)	105 (51.2)	< 0.001
Yes	1033 (80.3)	256 (98.1)	651 (98.9)	63 (38.7)	63 (30.7)	
Unknown	51 (4.0)	0 (0.0)	0 (0.0)	14 (8.6)	37 (18.0)	
*Radiotherapy*						
No	126 (9.8)	28 (10.7)	65 (9.9)	15 (9.2)	18 (8.8)	0.904
Yes	1161 (90.2)	233 (89.3)	593 (90.1)	148 (90.8)	187 (91.2)	
*Adjuvant chemotherapy*						
No	267 (20.7)	63 (24.1)	117 (17.8)	44 (27.0)	43 (21.0)	0.022
Yes	941 (73.1)	184 (70.5)	490 (74.5)	112 (68.7)	155 (75.6)	
Unknown	79 (6.1)	14 (5.4)	51 (7.8)	7 (4.3)	7 (3.4)	
*Recurrence*						
No	1226 (95.3)	250 (95.8)	638 (97.0)	144 (88.3)	194 (94.6)	< 0.001
Yes	61 (4.7)	11 (4.2)	20 (3.0)	19 (11.7)	11 (5.4)	
*Metastasis*						
No	1149 (89.3)	234 (89.7)	596 (90.6)	137 (84.0)	182 (88.8)	0.116
Yes	138 (10.7)	27 (10.3)	62 (9.4)	26 (16.0)	23 (11.2)	
*Death*						
No	1087 (84.5)	224 (85.8)	569 (86.5)	122 (74.8)	172 (83.9)	0.003
Yes	200(15.5)	37(14.2)	89(13.5)	41(25.2)	33(16.1)	

*N* number of patients, *IDC* Invasive ductal carcinoma, *MRM* modified radical mastectomy, *BCS* breast-conserving surgery

*Some HR− patients received hormone therapy in some different protocols during previous decades

As demonstrated in Table 1, clinicopathologic and treatment patterns differed by molecular subtypes. Patients with HR−/HER2+ status tended to have larger tumor size (*P* = 0.001), higher histologic grade (*P* < 0.001), higher stage (*P* = 0.038) and positive lymph node (*P* < 0.001). In contrast, smaller tumor size (*P* = 0.001), lower stage (*P* = 0.038) and lower grade (*P* < 0.001), were more common in HR+/HER2− patients. The lowest positive lymph nodes rate (about 50%) was also observed in triple-negative breast cancer (TNBC) patients (*P* < 0.001).

Almost half of women were treated by BCS; however, in HR−/HER2+ patients, MRM was the more prevalent procedure (*P* = 0.001). Hormone therapy differed significantly between subtypes. Virtually all HR-positive patients received hormone therapy compared with less than 40% of HR-negative patients. In addition, nearly all of the patients received radiotherapy and adjuvant chemotherapy, although in HER2− patients, adjuvant chemotherapy was more common (*P* = 0.022).

**Fig. 1:**
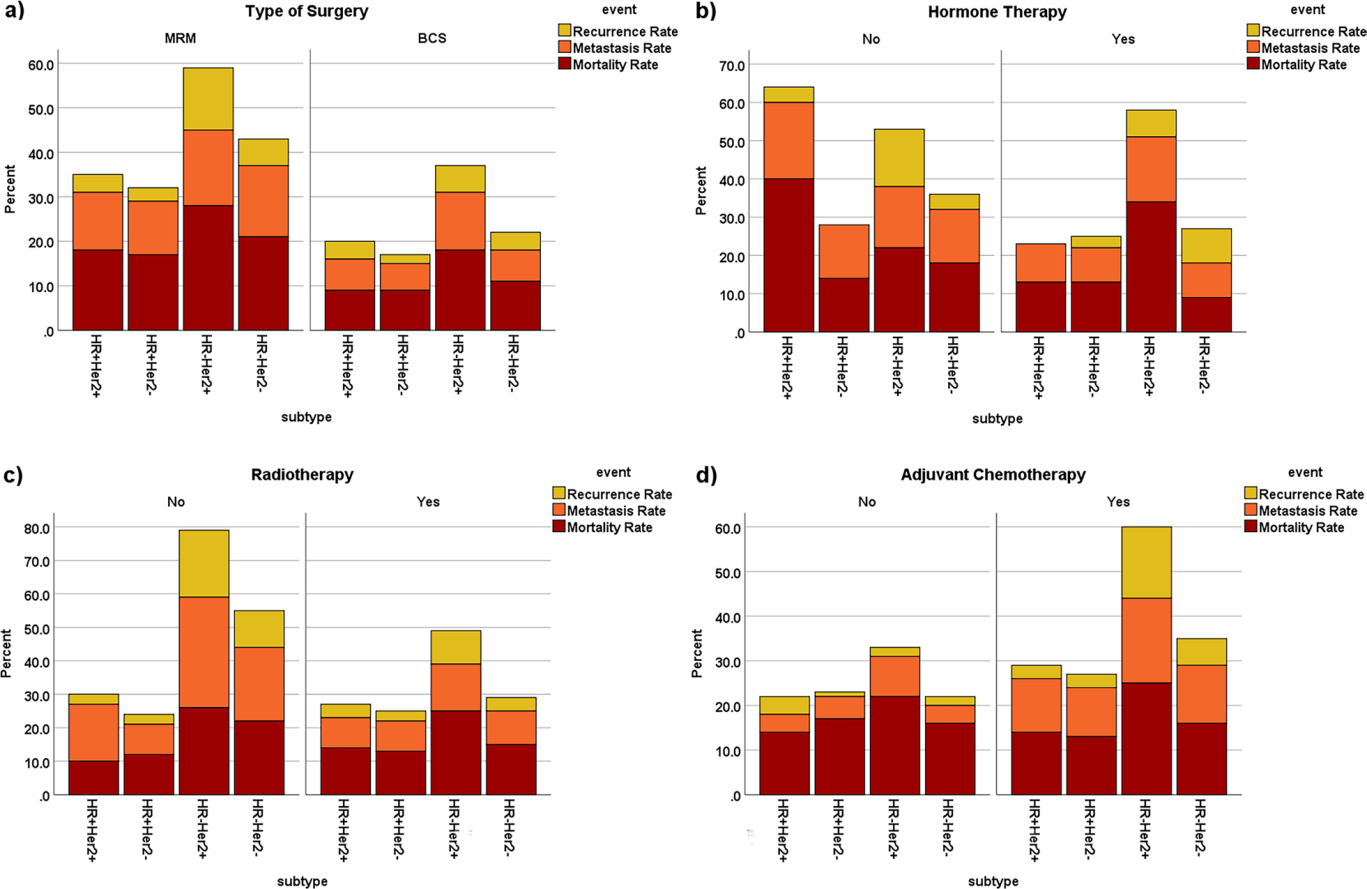
Recurrence, metastasis and mortality rates of breast cancer patients according to **a** type of surgery, **b** hormone therapy, **c** radiotherapy, **d** adjuvant chemotherapy

Figure 1 shows recurrence, metastasis and mortality rates by molecular subtypes according to treatment types. In all subgroups, patients who had MRM surgery experienced higher rate of mortality, metastasis, and recurrence than those who undergo BCS surgery (Fig. 1.a). Furthermore, patients who received hormone therapy, particularly HR+/HER2+ tumors, had lower mortality and metastasis rates, while HR−/HER2+ tumors had higher mortality rate (Fig. 1.b). The mortality rates of patients who received radiation therapy and/or adjuvant chemotherapy were nearly identical to those of other patients, as shown in Fig. 1.c and d. Patients with negative HR who received radiation therapy had a lower recurrence and metastasis rates, and those who received adjuvant chemotherapy had a greater recurrence and metastasis rate.

### Survival outcome

**Fig. 2:**
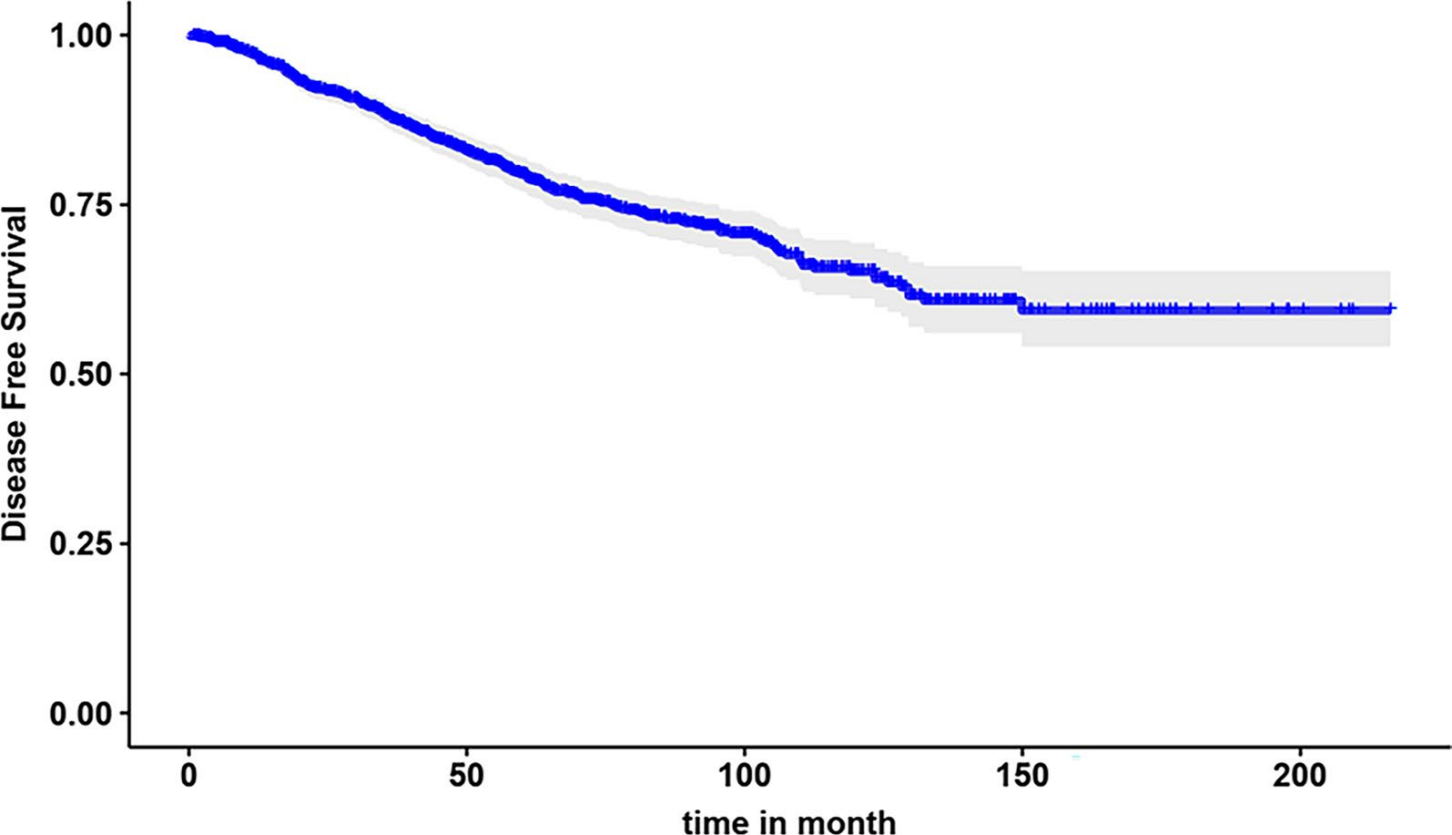
Kaplan–Meier disease-free survival curve with 95% CL in the month for breast cancer patient

The Kaplan–Meier method estimated 170.09 ± 3.11 months for mean OS and 155.2 ± 3.32 months for mean DFS. In addition, 1, 5, 7, 10, and 15-year OS rate was calculated 98%, 86%, 79%, 72%, and 67%, respectively. The Kaplan Meyer DFS curve with the 95% confidence interval is shown in Fig. 2.

The survival plot is almost flat after about 150 months. The cure fraction is estimated about 60% (the difference between the smooth sequence of the chart and the zero value on the survival probability axis). Patients who survived longer than 150 months were intuitively considered as cured or long-term survivors.

**Fig. 3:**
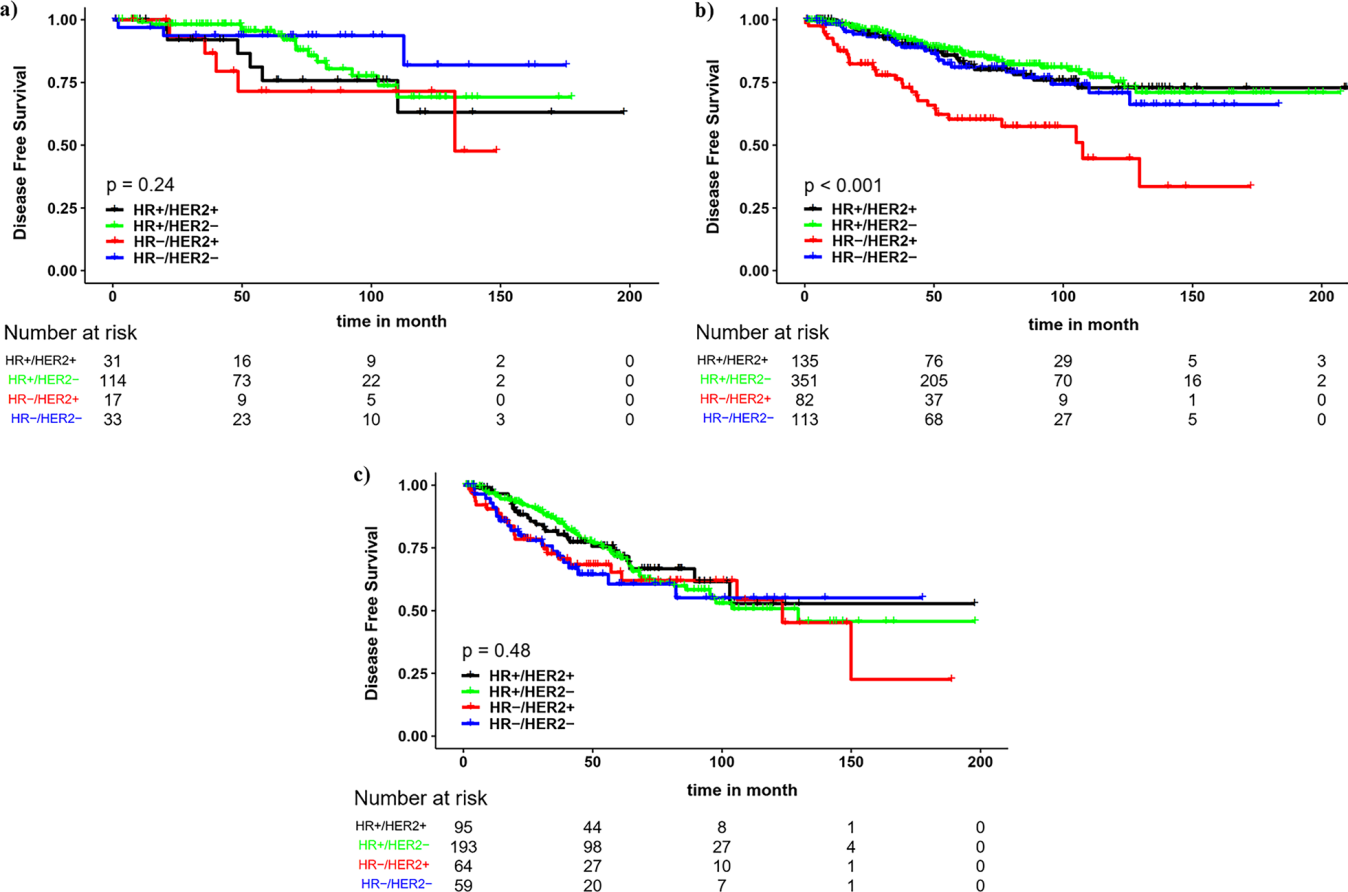
DFS of **a** stage I; **b** stage II; **c** stage III breast cancer by different molecular subtype

The 5-year DFS was 77.3% in HR+/Her2+, 84.2% in HR+/Her2−, 62.3% in HR−/Her2+, and 76.5% in TNBC (Table 2). In general, DFS of breast cancer subtypes were associated with tumor stage (Fig. 3). The DFS was not significantly different among molecular subtypes in stages I and III breast cancer (Fig. 3a and c). By contrast, in stage II, HR−/HER2+ patients had worst prognosis with 5-year DFS of 57.4% and HR+/HER2− patients had best DFS of 87.3%. The 5-year DFS estimated in stage II TNBC was 79.2%, which was lower than DFS estimated in the HR+/HER2− group (Table 2).

**Table 2:** The OS and DFS of breast cancer molecular subtypes in different stages of the disease

	N	5-Years outcome				
		Mortality rate N (%)	RR N (%)	DMR N (%)	OS	DFS
*All patients*	1287	134 (10.4)	53 (4.1)	82 (6.4)	86.3	79.3
HR+/Her2+	261	30 (11.5)	9 (3.4)	23 (8.8)	83.4	77.3
HR+/Her2−	658	47 (7.1)	17 (2.6)	47 (7.1)	90.2	84.2
HR−/Her2+	163	28 (17.2)	17 (10.4)	23 (14.1)	77.8	62.3
HR−/Her2−	205	29 (14.1)	10 (4.9)	19 (9.3)	81.0	76.5
*p-value*^*a*^					< 0.001	< 0.001
*Stage I*	195	3 (1.5)	8 (4.1)	6 (3.1)	96.8	89.3
HR+/Her2+	31	2 (6.5)	1 (3.2)	2 (6.5)	89.2 +	63.1
HR+/Her2−	114	1 (0.9)	2 (1.8)	2 (1.8)	97.0	93.8
HR−/Her2+	17	0 (0)	3 (17.6)	2 (11.8)	90.0	47.7
HR−/Her2−	33	0 (0)	2 (6.1)	0 (0)	90.0	81.9
*p-value*^*a*^					0.064	0.004
*Stage II*	681	56 (8.2)	30 (4.4)	55 (8.1)	88.6	82.2
HR+/Her2+	135	11 (8.1)	4 (3)	11 (8.1)	87.0	81.7
HR+/Her2−	351	16 (4.6)	11 (3.1)	21 (6)	92.8	87.3
HR−/Her2+	82	17 (20.7)	9 (11)	12 (14.6)	71.1	57.4
HR−/Her2−	113	12 (10.6)	6 (5.3)	11 (9.7)	83.9	79.2
*p-value*^*a*^					< 0.001	< 0.001
*Stage III*	411	75 (18.2)	15 (3.6)	51 (12.4)	75.8	68.6
HR+/Her2+	95	17 (17.9)	4 (4.2)	10 (10.5)	73.9	69.1
HR+/Her2−	193	30 (15.5)	4 (2.1)	24 (12.4)	78.3	70.9
HR−/Her2+	64	11 (17.2)	5 (7.8)	9 (14.1)	76.7	62.0
HR−/Her2−	59	17 (28.8)	2 (3.4)	8 (13.6)	57.1	55.1
*p-value*^*a*^					0.078	0.130

*N* number of patients, *RR* recurrence rate, *DMR* distant metastasis rate, *OS* overall survival, *DFS* disease free survival

^a^ log-rank test

### Prognostic factors of DFS survival

We performed the univariate and multivariate Cox mixture cure model to analyze the prognostic factors for short-term and long-term DFS of breast cancer patients, as shown in Table 3.

**Table 3:** Prognostic factors of breast cancer DFS using cox mixture cure model

Variable	Univariate				Multivariate			
	HR (95% CI)	*P*-value	OR (95% CI)	*P*-value	HR (95% CI)	*P*-value	OR (95% CI)	*P*-value
*Age*								
< 50	Ref		Ref		Ref		Ref	
≥ 50	1.27 (1,1.61)	0.049	0.78 (0.62,0.99)	0.037	0.8 (0.53,1.22)	0.302	0.81 (0.53,1.23)	0.319
*BMI*								
< 30	Ref		Ref		Ref		Ref	
≥ 30	1.08 (0.85,1.38)	0.512	0.45 (0.36,0.58)	< 0.001	0.94 (0.72,1.23)	0.654	0.41 (0.3,0.56)	< 0.001
*Marital status*								
Single	Ref		Ref		Ref		Ref	
Married	0.87 (0.69,1.1)	0.242	1.56 (1.18,2.07)	0.002	1.28 (0.93,1.76)	0.131	1.62 (1.14,2.3)	0.007
*Education level*								
< diploma	Ref		Ref		Ref		Ref	
≥ diploma	1.27 (0.95,1.71)	0.106	1.82 (1.46,2.28)	< 0.001	0.7 (0.53,0.91)	0.009	1.13 (0.85,1.49)	0.390
*Family history*								
No	Ref		Ref		Ref		Ref	
Yes	0.58 (0.41,0.82)	0.002	0.48 (0.34,0.66)	< 0.001	0.94 (0.64,1.37)	0.747	0.66 (0.45,0.99)	0.042
*Menopausal status*								
Premenopausal	Ref		Ref		Ref		Ref	
Postmenopausal	1.35 (1.07,1.72)	0.013	0.79 (0.62,0.99)	0.042	1 (0.66,1.53)	0.998	0.63 (0.41,0.96)	0.031
*Pathology*								
Others	Ref		Ref		Ref		Ref	
Inv. Ductal Ca	0.89 (0.59,1.34)	0.565	0.56 (0.39,0.82)	0.003	0.68 (0.4,1.15)	0.145	0.26 (0.15,0.44)	< 0.001
*Tumor size (cm)*								
< 2	Ref		Ref		Ref		Ref	
2–5	1.97 (1.41,2.76)	< 0.001	0.99 (0.75,1.31)	0.968	1.39 (0.85,2.28)	0.195	1.13 (0.68,1.88)	0.644
> 5	3.2 (2.22,4.62)	< 0.001	0.67 (0.48,0.93)	0.015	1.99 (1.1,3.6)	0.023	1.86 (0.96,3.57)	0.064
*Tumor grade*								
1	Ref		Ref		Ref		Ref	
2	0.8 (0.56,1.14)	0.214	0.63 (0.44,0.92)	0.015	1.04 (0.7,1.55)	0.855	1.15 (0.75,1.75)	0.521
3	1.51 (1.03,2.23)	0.036	0.59 (0.39,0.88)	0.010	1.35 (0.86,2.12)	0.190	0.93 (0.57,1.52)	0.776
*Stage*								
I	Ref		Ref		Ref		Ref	
II	1.94 (1.29,2.93)	0.002	1.13 (0.81,1.58)	0.460	1.04 (0.52,2.05)	0.921	1.3 (0.67,2.51)	0.433
III	2.22 (1.47,3.35)	< 0.001	0.45 (0.32,0.65)	< 0.001	0.49 (0.22,1.12)	0.091	0.21 (0.09,0.48)	< 0.001
*LN metastasis*								
No	Ref		Ref		Ref		Ref	
Yes	1.5 (1.15,1.96)	0.003	0.53 (0.41,0.67)	< 0.001	1.58 (1.04,2.39)	0.031	1.15 (0.76,1.74)	0.510
*Molecular subtype*								
HR+/Her2−	Ref		Ref		Ref		Ref	
HR+/Her2+	1.53 (1.11,2.12)	0.010	1.17 (0.87,1.58)	0.295	1.34 (0.95,1.89)	0.098	1.27 (0.9,1.8)	0.177
HR−/Her2+	1.38 (1.01,1.89)	0.045	0.38 (0.27,0.55)	< 0.001	0.91 (0.6,1.39)	0.654	0.22 (0.13,0.38)	< 0.001
HR−/Her2−	1.78 (1.26,2.5)	0.001	1.28 (0.92,1.78)	0.139	1.5 (0.94,2.39)	0.091	1.08 (0.65,1.81)	0.767
*Type of surgery*								
MRM	Ref		Ref		Ref		Ref	
BCS	0.57 (0.45,0.73)	< 0.001	1.51 (1.21,1.89)	< 0.001	0.55 (0.41,0.75)	< 0.001	0.97 (0.71,1.35)	0.876
*Hormone therapy*								
No	Ref		Ref		Ref		Ref	
Yes	0.67 (0.51,0.9)	0.007	1.34 (0.99,1.81)	0.059	1.04 (0.7,1.56)	0.848	0.98 (0.59,1.62)	0.931
*Radiotherapy*								
No	Ref		Ref		Ref		Ref	
Yes	1.15 (0.79,1.67)	0.479	0.89 (0.61,1.3)	0.553	1.1 (0.68,1.79)	0.699	0.92 (0.54,1.56)	0.761
*Adjuvant chemotherapy*								
No	Ref		Ref		Ref		Ref	
Yes	0.61 (0.44,0.83)	0.002	1.1 (0.83,1.45)	0.499	0.55 (0.39,0.78)	0.001	0.76 (0.53,1.1)	0.144

*HR* hazard ratio, *OR* odds ratio, *CI* confidence interval, *MRM* modified radical mastectomy, *BCS* breast-conserving surgery

The effect of factors on survival time and cure probability of patients were indicated by HR and OR, respectively. If the HR is more/less than one, it means that the variable is a risk/protective factor for survival. Whereas the OR more/less than one means that the variable is a protective/risk factor for odds of cure. The greater distance from one, the greater effect.

In univariate analysis, the hazard ratio of DFS in the TNBC, HR+/HER2+, and HR−/HER2+ were 1.78, 1.53 and 1.38 compared to HR+/HER2 as the reference subtype. However, only the HR−/Her2+ subtype was associated significantly with the cure probability (OR = 0.38). On the other hand, after adjusting for demographic and clinical factors, the risk of mortality, metastasis, and recurrence was not significantly associated with molecular subtype in the short-term. Still, the cure probability of HR−/Her2+ patients was significantly lower than HR+/HER2− patients (OR = 0.22).

Other associated factors with worse short-term DFS included larger tumor size (HR = 1.99) and positive lymph nodes (HR = 1.58). Furthermore, high-level of education (HR = 0.7), BCS surgery (HR = 0.55), and adjuvant chemotherapy (HR = 0.55) had a protective effect on DFS.

In the long-term, married women were more likely to be cured (OR = 1.62). Regardless, the cure probability of the women with obesity (OR = 0.41), postmenopausal status (OR = 0.63), family history of cancer (OR = 0.66), IDC pathology (OR = 0.26), and advanced tumor stage (OR = 0.21) was significantly lower than the others.

## Discussion

In modern medicine, predicting the patient's prognosis is essential to avoid overtreatment or undertreatment [17]. In the present study, molecular subtypes were used to assess the breast cancer patients' survival by mixture cure model. Recognition separately the different covariate effects on cure probability and survival of patients can be considered as the advantages of this model.

In our study, the mean age of patients was 47.00 ± 10.72 years, which was similar to the mean age previously reported in a meta-analysis of 24 Iranian breast cancer studies [18] and a review conducted among Asian countries [19]. In addition, based on our results, the proportion of patients younger 50 in Iran was much higher than in high-income countries, such as the USA [20], Germany [21], and the Netherlands [22]. Furthermore, there were 26% of young patients (less than 40 years old) with breast cancer, while most other countries had a smaller proportion of young onset patients[23, 24]. This discrepancy might be related to differences in disease patterns or age distributions; the median age of women in Iran and Europe are 32 and 45.5 years, respectively [25]. Accordingly, this result emphasizes the value of local data in developing national mammography guidelines that recommend starting women screening before the age of 50. Our results revealed no significant relationship between diagnosis age and breast cancer patients' survival rate or cure probability. However, some studies have reported significant association [26–28].

HR+/HER2− was the most frequent subtype, with a prevalence of 51.1%, which is lower than those reported for developed countries such as Canada (64.8%) [11] and the USA (66.6%) [12]. In this study, the frequency of HR+/HER2+, HR−/HER2+, and TNBC were 20.3%, 12.7%, and 15.9%, respectively. Other studies from the USA (6% HR−/HER2+; 17.4% TNBC) [29] and China (6.8% HR−/HER2+; 18.3% TNBC) [30] reported lower prevalence of HR−/HER2+ but almost the same for TNBC. Furthermore, a prior study in Iran reported a prevalence of 61% in HR+/HER2−, 8.1% in HR−/HER2+, and 23% for the TNBC [31]. Several factors can cause disparities in our results, including differences in age distribution, genetic variation, or classification criteria for molecular subtypes.

The 5-year OS rate was 86.3% for all patients. Before 2000, the survival rate of Japanese, Korean, Turkish, and Arab females was 88.1%, 83.7%, 76.7%, and 64.5%, respectively [32]. Moreover, it is slightly lower than the 5-year OS of 91% in the USA [33] and 89.7% in Brazil [34]. Considering breast cancer research center as a referral center in Iran, it seems that the 5-year survival rate in Iran is higher than it in many Asian counterparts but lower than in American countries. It can be concluded that even without a national screening policy and lack of availability to some new therapeutic modalities, our survival rate is within an acceptable limit, so it is expected to improve soon.

DFS and OS rates significantly differ between subtypes based on Log-Rank test, ranging from highest to lowest HR+/HER2− (90.2% OS; 84.2% DFS), HR+/HER2+ (83.4% OS; 77.3% DFS), TNBC (81.0% OS; 76.5% DFS), and HR−/HER2+ (77.8% OS; 62.3% DFS), respectively; which was confirmed by some other studies [30, 31]. HR-positive tumors showed better survival compared to HR-negative tumors. In addition, we detected a relevant difference in OS and DFS according to breast cancer subtypes in stages I-III disease. In stages I and II, 5-year DFS of HR−/HER2+ patients were significantly lower than others. Stage III of disease generally had the worst prognosis, and the difference between subtypes in these patients was not statistically significant. DFS of the HR+/Her2− subtype was high in all stages, in line with finding in America and Europe [35–37]. From the above results, comparing subtypes at different stages of the disease can provide more accurate information.

According to our study, the HR−/HER2+ subtype had the worst prognosis. Even though some studies have found similar results [30, 31], but it differ from the common belief that TNBC has the worst prognosis [37–39]. The reasons might be the larger tumor size, more advanced stage, more positive lymph node, or poor treatment in these patients. Newer HER2-directed therapies, such as pertuzumab and trastuzumab, as well as T-DM1 and TKI, may improve HER2+ patients' outcomes [40].

This research revealed that the tumor characteristics and survival rates varied with the molecular subtype. Like other studies, more proportion of tumors under 2 cm, stage I, and low histologic grade were in HR+/HER2− patients [30, 31]. By contrast, HR−/HER2+ tumors tended to be larger in size, higher stage, higher histologic grade, and positive lymph nodes. Even though these results differ from some earlier studies that reported worse tumor features in TNBC [38, 41], they are consistent with those of Chinese [30] and Canadians [12] that observed the worst tumors in HR−/HER+ patients. As suggested in the literature [31, 42], breast cancer patients with TNBC have fewer positive lymph nodes, increasing the possibility of blood spreading of this cancer than lymphocytes. Future researches are needed to clarify this molecular subtypes' mechanism.

The results of multivariate analysis showed that several clinicopathological factors such as larger tumor size, IDC pathology and more advanced stage of the disease are correlated with breast cancer survival. Some other studies have emphasized the importance of these factors [30, 38]. Furthermore, the nodal status remains one of the most essential risk factors for survival and metastasis as we also showed in our study. Therefore, node status is also an important determinant in the decision-making for breast cancer treatment. Obesity, menopause, and a family history of cancer were also identified as risk factors for patients' survival, while higher education, adjuvant chemotherapy, BCS, and marital status were protective. So, it seems that postmenopausal women and those with a family history of cancer should be given more attention in screening policies. Changing lifestyle and diet modification can also help improve patients' survival.

After adjusting for pathological and demographic variables, short-term survival was not significantly different between the molecular subtypes, but in the long-term, the cure probability of HR−/HER2+ patients was much lower than the others. In other words, the short-term survival differences between subtypes are mostly due to pathological and demographic variables. However, even after adjusting for these variables, HR−/HER2+ patients still are less likely to be cured in the long-term.

The current study represents the biomarker analysis of DMR and RR in breast cancer by molecular subtype. For both DMR and RR, patients with HR+/HER2− tumors had the most favorable prognosis, with DMR and RR of only 7.1% and 2.6% at 5 years. Conversely, HER2 positive and TNBC exhibited the highest rates of DMR (14.1% and 9.3%) and RR (10.4% and 4.9%). Our results have several similarities with a retrospective cohort study of Asian young breast cancer patients that found HR−/HER2+ tumors had the highest recurrence and metastasis rates. In contrast, HR+/HER2− tumors displayed the lowest LRR [30]. Another study of 10-year recurrence in European breast cancer patients announced that those with HR−/HER2+ and TNBC had a significantly higher recurrence and metastasis rate than HR-positive profiles, which was in agreement with our findings [22]. A Canadian study showed that, after a median follow-up of 6.9 years, 8% of HER2-positive cancers had a local recurrence, compared to 12% in TNBC. Moreover, they showed that distant metastases occurred in 19.2% of HER2-positive and 27.4% of TNBC. They also noted that the HR+ patients had the best prognosis of local recurrence and distant metastasis [43].

In this study, we tried to predict breast cancer patients' short- and long-term survival with robust statistical methods. We have some limitations in our study. First, we classified molecular subtypes based on HR and HER2 status without additional biomarkers, including Ki-67 and cytokeratin levels. Second, the medical records we used did not include detailed information on different therapies, such as endocrine therapies, HER2-directed therapies, or chemotherapy. By adjusting for these factors, we might reduce confounding effects and improve our knowledge about the survival of breast cancer molecular subtypes. Third, since there was a large amount of missing data, especially for ER, PR and HER2, we had to use the limited number of data available. So, the results should be interpreted with caution.

## Conclusion

In conclusion, our study showed that HR+/HER2− breast cancer is the most prevalent tumor subtype, similar to other countries. It had the best prognosis, followed by HR+/HER2+, triple-negative, and HR−/HER2+ patients. Based on the mixture cure model, prognostic variables showed different relations with the DFS in the short-term and cure probability in the long-term. Accordingly, even after controlling for variables such as tumor stage, tumor size, positive lymph nodes, and obesity, HR−/HER2+ patients still had a lower cure probability in the long time. Starting screening women at a younger age and shorter intervals to detect patients in the early stages and using new targeted therapies based on molecular subtypes may improve breast cancer patients' survival and cure probability.

## Data Availability

The datasets generated and analyzed during the current study are not publicly available due to containing information that could compromise the privacy of research participants, but are available from the corresponding author on reasonable request.

## Abbreviations

N: Number of patients
IDC: Invasive ductal carcinoma
MRM: Modified radical mastectomy
BCS: Breast-conserving surgery
RR: Recurrence rate
DMR: Distant metastasis rate
OS: Overall survival
DFS: Disease free survival
HR: Hazard ratio
OR: Odds ratio
CI: Confidence interval
ER: Estrogen receptors
PR: Progesterone receptors
HER-2: Human epidermal growth factor receptor-2
HR: Hormone receptors
FISH: Fluorescence in situ hybridization
TNBC: Triple-negative breast cancer
